# Supplementary material for: Sexual selection for both diversity and repetition in birdsong
Source: Nat Commun. 2023 Jun 16;14:3600. doi: 10.1038/s41467-023-39308-5 (PMC10275917; doi:10.1038/s41467-023-39308-5)
Supplement: Supplementary file 1 — Supplementary Information [file 41467_2023_39308_MOESM1_ESM.pdf]

Sexual selection for both diversity and repetition in birdsong

Javier Sierro, Selvino R. de Kort & Ian R. Hartley

Supplementary Information

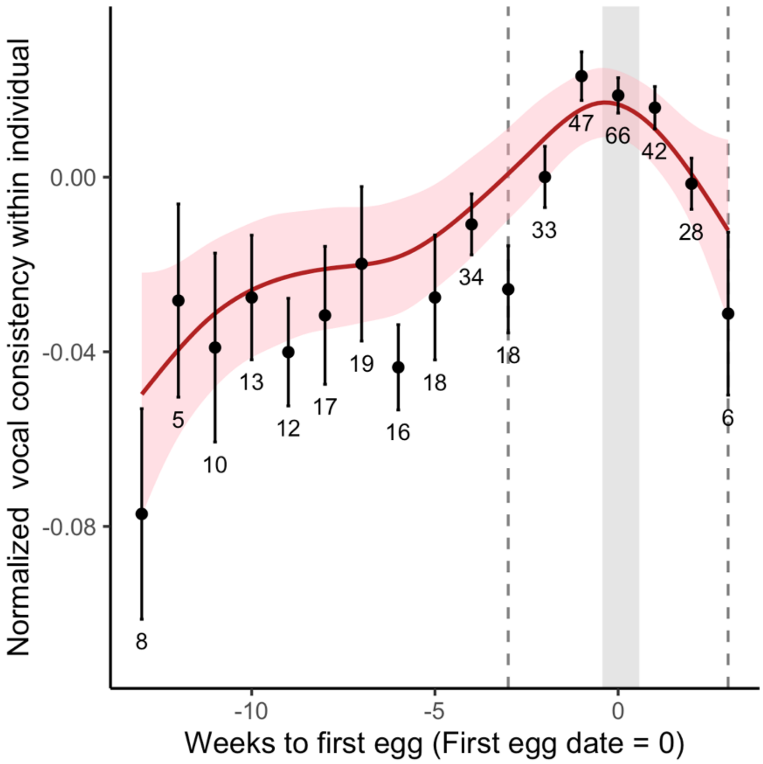

Figure S1 – Seasonal variation in male blue tit vocal consistency, normalized within individual, at a weekly level from Winter (January) to Spring (May), shown in relation to the laying of the first egg for each male (week of first egg = 0). Points show the mean of the normalized (centred but not scaled) vocal consistency of all individuals per week, with the associated error bars showing the SE of vocal consistency within that week for all individuals. The line and the shaded area show the predicted values obtained from a GAMM with the associated 95% CI. The vertical dashed lines delimit the breeding period shown in Figure 2A. The greyed area shows the female receptive period.

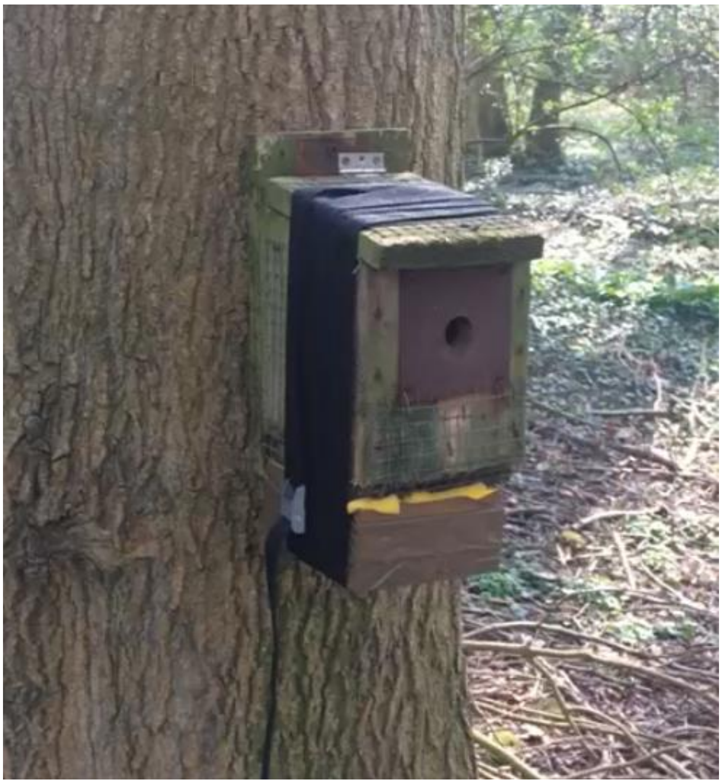

Figure S2 – Photo of the experimental set up on the nest box, with the recording device placed inside the box, attached to the underside of the lid, and the speaker enclosed inside an insulating box and strapped to the bottom panel of the nest box.

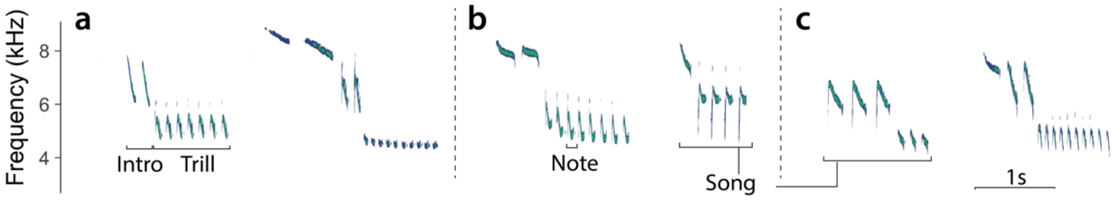

Figure S3 – Spectrograms of three different pairs of song types (a-c) collected from three different male blue tits. Each pair was used to build one playback stimulus for the female choice playback experiment.

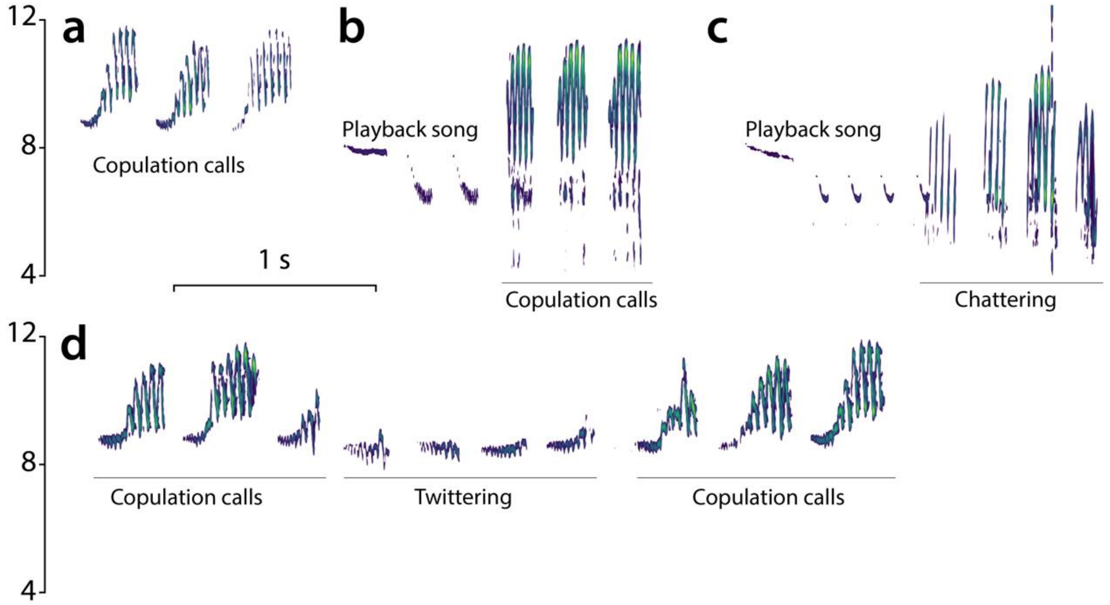

Figure S4 – **Spectrogram of female blue tit copulation solicitation calls, chattering calls and twittering calls recorded inside the nest box from three different females**. Calls in (**a**) are typical copulation calls [24,6]. Calls in (**b**) and (**c**) are copulation and chattering calls respectively, recorded from the same female in the same trial, showing variation in frequency range. In (**d**), copulation calls gradually transform into twittering and vice versa. Context, acoustic structure and delivery mode seem very similar in all three call types, varying along a gradient of frequency range and amplitude of the frequency modulation.

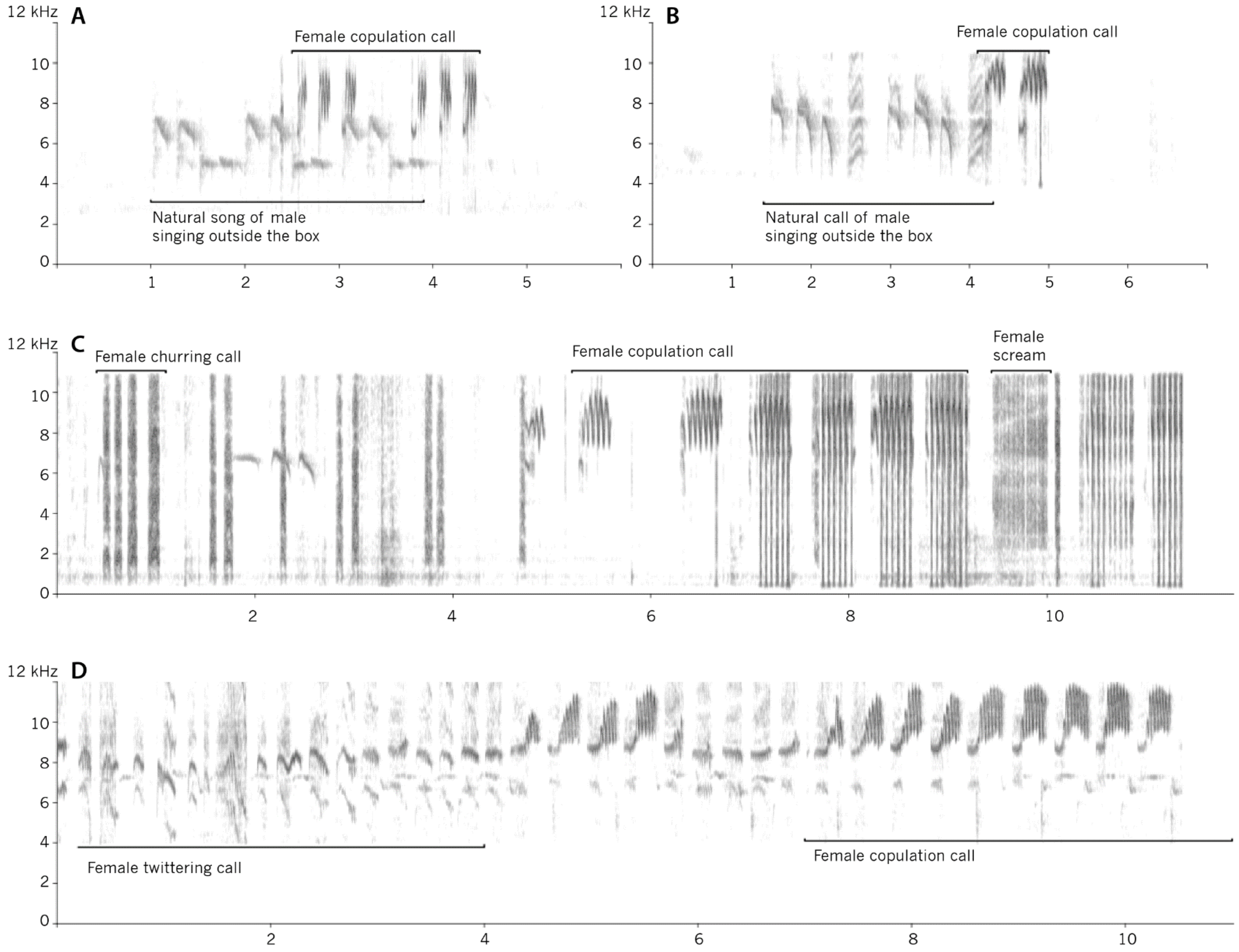

Figure S5 – Spectrograms of natural vocal response of female blue tits inside the nest, without playback presentation. (**a**) - (**b**) show the natural vocal interaction between the female, inside the nest, and her male partner, outside the nest. Figure (**c**) and (**d**) show two complete copulation solicitation displays characterized by high rate production of loud copulation calls, recorded in two different nest boxes.

Table S1 – Estimates of the full model investigating impact of male song traits on reproductive success (clutch size).

Fixed effects					Random effects	
**Variable**	**Estimate**	**T value**	**2.5% CI**	**97.5% CI**	**Variable**	**Variance**
Intercept	9.588	35.336	9.067	10.11	Individual	0.62 (0.79)
Vocal consistency	0.359	2.551	0.089	0.628	Residual	1.35 (1.16)
Repertoire size	0.073	0.497	-0.208	0.356		
Age of female	0.063	0.464	-0.197	0.323		
Year:2019	-0.738	-1.894	-1.483	0.007		
Year:2020	-1.686	-3.874	-2.519	-0.851		
Date of first egg (Julian date per year)	-1.04	-5.716	-1.388	-0.693		

For each fixed effect we show the model estimate, representing the size and direction of the effect, the T statistic and the 95% CI around the estimate. Vocal consistency had a positive significant effect on clutch size as the 95% CI do not overlap with zero. The estimated variance explained by the random effects is shown in the last two columns.

Table S2 – List of models investigating the variation in reproductive success with male song traits

Intercept	Year	First egg date	Female age	Male song-type repertoire	Male vocal consistency	df	logLik	AICc	delta	Weight
9.597	+	-1.039			0.367	7	-185.01	385.163	0	0.598
9.681	+	-1.086				6	-187.34	387.528	2.365	0.183
9.591	+	-1.036		0.080	0.365	8	-185.87	389.223	4.06	0.078
9.593	+	-1.044	0.070		0.36	8	-185.96	389.411	4.248	0.071
9.674	+	-1.091	0.102			7	-188.13	391.401	6.238	0.026
9.673	+	-1.083		0.089		7	-188.144	391.427	6.264	0.026

List of models computed during the model selection process for the analysis investigating the variation in reproductive success with male song traits. Models with an ΔAICc < 7 are shown. Final model was the average model of those with an ΔAICc < 2, which in this case is only the first, best model.

Table S3 – Results from the GAMM model fitted to investigate seasonal variation of vocal consistency (normalized within individual) as a function of weeks in relation to the first egg date and singing context.

**SMOOTH TERMS**				
**Fixed effect**	**EDF**	**F**	**P value**	
Weeks to first egg	4.52	10.92	< 0.0001	
**PARAMETRIC TERMS**				
**Fixed effect**	**Estimate**	**T**	**2.5% CI**	**97.5% CI**
Intercept	-0.008	-2.8	-0.013	-0.003
Weeks to first egg (week of first egg = 0)	-0.069	-5.26	-0.095	-0.043
Context (dawn song vs. day-time song)	0.021	4.07	0.011	0.031

The coefficient of the smooth term shows that the change of vocal consistency in relation to weeks to first egg is not linear, with Effective Degrees of Freedom (EDF) higher than 1. Test statistics are derived from the frequentist properties of Bayesian confidence intervals for smooths (Marra & Wood 2012). Furthermore, the seasonal change in vocal consistency is significant, as shown by the P value of the EDF. The second part of the table shows the parametric estimates for the predictors, indicating the vocal consistency at the intercept (the start of the season). The linear coefficient for weeks to first egg is not significant but vocal consistency was significantly higher during dawn song than during day-time singing.

Table S4 – Results from the GAMM model fitted to investigate seasonal variation of vocal consistency during the breeding period.

**SMOOTH TERMS**						
**Fixed effect**	**EDF**	***F***	***P***			
Days to first egg	6.56	13.1	< 0.001			
**PARAMTRIC TERMS**						
**Fixed effect**					**Random effects**	
**Variable**	**Estimate**	***T***	**2.5% CI**	**97.5% CI**	**Variable**	**Variance (SD)**
Intercept	0.850	188.8	0.836	0.854	Individual	0.0014 (0.038)
Days to first egg (first egg date = 0)	-0.010	-1.19	-0.028	0.007	Smooths	$2.6*10^{-5}$ (0.0052)
Context (dawn song vs. day-time song)	0.015	5.52	0.01	0.02	Residual	0.0053 (0.073)

The coefficient of the smooth term shows that the change of vocal consistency in relation to days to first egg is not linear, with Effective Degrees of Freedom (EDF) higher than 1. The seasonal change in vocal consistency is significant, as shown by the P value of the EDF. Test statistics are derived from the frequentist properties of Bayesian confidence intervals for smooths (Marra & Wood 2012). The second part of the table shows the parametric estimates for the predictors, indicating that vocal consistency at the intercept is significantly higher than zero, as shown by the CI that do not overlap zero. However, the linear coefficient for days to first egg is not significantly different from zero, as shown by the CI overlapping zero. The parametric estimates show that context has a significant effect on vocal consistency as songs during dawn chorus are significantly more consistent than during day time singing. The estimated variance and the standard deviation (SD) explained by the random effects is shown in the last two columns.

Table S5 – Comparison of breeding behaviour between 13 females included in the experiment and 58 females excluded from the experiment within the same population, using two-sided, Mann-Whitney U tests. Per group, mean ± SD

Breeding data	Females included the experiment	Females excluded from the experiment	W	P
Clutch size	8.4 ± 1.4 eggs per nest	8.2 ± 1.8 eggs per nest	540	0.54
Brood size	5.9 ± 1.4 chicks per nest	4.8 ± 1.8 chicks per nest	593	0.13
Fledging success	5.3 ± 3.2 fledglings per nest	4.5 ± 3.2 fledglings per nest	560	0.33

Table S6 – Estimates of the full model investigating impact of male song traits on female vocal response.

**Fixed effects**					**Random effects**	
**Variable**	**Estimate**	***Z***	**2.5% CI**	**97.5% CI**	**Variable**	**Variance (SD)**
Intercept	-1.406	-5.87	-1.945	-0.911	Individual	0.46 (0.68)
Song rate	0.201	0.814	-0.283	0.717		
Vocal consistency	0.475	2.955	0.166	0.805		
Trill length (notes)	0.337	1.312	-0.196	0.856		

For each fixed effect we show the model estimate, representing the size and direction of the effect, the Z statistic and the 95% CI around the estimate. Vocal consistency had a positive significant effect on female vocal response as the 95% CI do not overlap with zero. The estimated variance and SD explained by the random effects is shown in the last two columns.

Table S7 – List of models computed during the model selection process for the analysis investigating the variation in female vocal response with playback song traits. All models with an ΔAICc < 7 are shown. Final model was the average model of those with an ΔAICc < 2.

Intercept	Trill length	Song rate	Vocal consistency	df	logLik	AICc	delta	Weight
-1.407			0.492	3	-52.351	111.793	0	0.55
-1.403	0.212		0.482	4	-51.867	113.638	1.845	0.219
-1.407		0.025	0.492	4	-52.344	114.593	2.8	0.136
-1.406	0.337	0.201	0.475	5	-51.535	116.071	4.278	0.065

Table S8 - Operational definitions of playback structure

**Playback structure**	**Definition**
Playback song-type bout	Portion of the playback stimulus that lasts 1:15 min and presents several repetitions of a single song type. Each repetition is one song and the number of songs per minute (song rate) is based on the song rate measured on 10 consecutive songs in the original recording
Playback Song bout	A larger portion of the playback stimulus that includes two consecutive song-type bouts of 1:15 min, hence it lasts 2:30 min
Playback Silence bout	A portion of playback that is silent and lasts 2:30 min
Playback round	A song bout and a silence bout together make a playback round (5 min). A complete trial consisted in the presentation of 12 playback rounds. The order of presentation of song and silence within playback round was randomized.

References for Supporting information

Marra G, Wood SN. Coverage properties of confidence intervals for generalized additive model components. *Scandinavian Journal of Statistics* **39**, 53-74 (2012).
