# Supplementary material for: Sexual selection for both diversity and repetition in birdsong
Source: Nat Commun. 2023 Jun 16;14:3600. doi: 10.1038/s41467-023-39308-5 (PMC10275917; doi:10.1038/s41467-023-39308-5)
Supplement: Supplementary file 3 — Description of Additional Supplementary Files [file 41467_2023_39308_MOESM3_ESM.pdf]

Description of Additional Supplementary Files

File Name: Supplementary Data 1
Description: This file includes all raw data necessary to conduct the statistical analysis presented in this study. The corresponding R code is also included in this file

File Name: Supplementary Data 2

Description: Contains the source data files used in each figure presented in the manuscript.

Figure 2a corresponds with "source.data.file.fitness.txt" and "source.data.file.fitness.fitted.txt"
Figure 2b corresponds with "source.data.file.fitness.perclutch.txt"
Figure 2c corresponds with "source.data.file.season.txt" and "source.data.file.season.fitted.txt"
Figure 2d corresponds with "source.data.file.dawn.context.txt"

Figure 3 corresponds with "source.data.file.warmupeffect.txt" and "source.data.file.warmupeffect.fitted.txt"

Figure 4a corresponds with "source.data.file.femchoice.data1.txt"
Figure 4b corresponds with "source.data.file.femchoice.data2.txt" and "source.data.file.femchoice.data2.fitted.txt"

Figure 5 corresponds with "source.data.file.habituation.data.txt" and "source.data.file.habituation.data.fitted.txt
