# Supplementary material for: Sexual selection for both diversity and repetition in birdsong
Source: Nat Commun. 2023 Jun 16;14:3600. doi: 10.1038/s41467-023-39308-5 (PMC10275917; doi:10.1038/s41467-023-39308-5)
Supplement: Supplementary file 6 — Reporting Summary [file 41467_2023_39308_MOESM6_ESM.pdf]

nature portfolio

Corresponding author(s): Javier Sierro, Selvino de Kort, Ian Hartley

Last updated by author(s): Apr 26, 2023

Reporting Summary

Nature Portfolio wishes to improve the reproducibility of the work that we publish. This form provides structure for consistency and transparency in reporting. For further information on Nature Portfolio policies, see our Editorial Policies and the Editorial Policy Checklist.

Statistics

For all statistical analyses, confirm that the following items are present in the figure legend, table legend, main text, or Methods section.

n/a	Confirmed	
☐	☒	The exact sample size (*n*) for each experimental group/condition, given as a discrete number and unit of measurement
☐	☒	A statement on whether measurements were taken from distinct samples or whether the same sample was measured repeatedly
☐	☒	The statistical test(s) used AND whether they are one- or two-sided<br>*Only common tests should be described solely by name; describe more complex techniques in the Methods section.*
☐	☒	A description of all covariates tested
☐	☒	A description of any assumptions or corrections, such as tests of normality and adjustment for multiple comparisons
☐	☒	A full description of the statistical parameters including central tendency (e.g. means) or other basic estimates (e.g. regression coefficient) AND variation (e.g. standard deviation) or associated estimates of uncertainty (e.g. confidence intervals)
☐	☒	For null hypothesis testing, the test statistic (e.g. *F*, *t*, *r*) with confidence intervals, effect sizes, degrees of freedom and *P* value noted<br>*Give P values as exact values whenever suitable.*
☒	☐	For Bayesian analysis, information on the choice of priors and Markov chain Monte Carlo settings
☐	☒	For hierarchical and complex designs, identification of the appropriate level for tests and full reporting of outcomes
☐	☒	Estimates of effect sizes (e.g. Cohen's *d*, Pearson's *r*), indicating how they were calculated

Our web collection on statistics for biologists contains articles on many of the points above.

Software and code

Policy information about availability of computer code

Data collection	No software was used for data collection
Data analysis	Audacity software (3.2.2), R software (4.2.1). R packages: seewave (2.2.0), tuneR (1.4.0), lme4 (1.1-30), MuMIn (1.47.1), gamm4 (0.2-6), stringr (1.4.1), dplyr (1.0.10) & ggplot2 (3.3.6)

For manuscripts utilizing custom algorithms or software that are central to the research but not yet described in published literature, software must be made available to editors and reviewers. We strongly encourage code deposition in a community repository (e.g. GitHub). See the Nature Portfolio guidelines for submitting code & software for further information.

Data

Policy information about availability of data

All manuscripts must include a data availability statement. This statement should provide the following information, where applicable:

- Accession codes, unique identifiers, or web links for publicly available datasets
- A description of any restrictions on data availability
- For clinical datasets or third party data, please ensure that the statement adheres to our policy

The datasets used in this study are available as supplementary material of this publication

Research involving human participants, their data, or biological material

Policy information about studies with human participants or human data. See also policy information about sex, gender (identity/presentation), and sexual orientation and race, ethnicity and racism.

Reporting on sex and gender	n/a
Reporting on race, ethnicity, or other socially relevant groupings	n/a
Population characteristics	n/a
Recruitment	n/a
Ethics oversight	n/a

Note that full information on the approval of the study protocol must also be provided in the manuscript.

Field-specific reporting

Please select the one below that is the best fit for your research. If you are not sure, read the appropriate sections before making your selection.

☐ Life sciences ☐ Behavioural & social sciences ☒ Ecological, evolutionary & environmental sciences

For a reference copy of the document with all sections, see nature.com/documents/nr-reporting-summary-flat.pdf

Ecological, evolutionary & environmental sciences study design

All studies must disclose on these points even when the disclosure is negative.

Study description	The study includes observational data and empirical data. Observational data includes song recordings made in the field from wild birds. The experiment was a playback experiment also conducted under field conditions, testing female preference for song traits. We then conducted a detailed analysis of acoustic structure of birdsong in the lab
Research sample	Samples are different depending on which part of the study. For the observational data, we collected data on 99 male blue tits over three years, with more than 7,000 songs analysed. Then, subsets were taken for the analysis of different aspects of song variation. The experiment was conducted on 15 individual females, although the final data set was reduced to 13 trials on 13 females.
Sampling strategy	Sampling was conducted by walking transects through the woodland. We aimed to collect as many recordings as possible from all males in the population. For the playback experiment, sampling strategy involved random selection of nests in our population. In this case, sample size was limited by the duration of the fertile period, as we could only conduct one or two trials a day for a period of about 9 days. To increase statistical power, we took repeated measurements of the response from the same individuals within trials.
Data collection	Data was collected by two observers in the field, recording song data using Sennheiser ME67 microphone and a Marantz PMD661, and breeding data on a wild population of blue tits. We used Nikon Prostaff 8x42 binoculars for identification of birds in the field.
Timing and spatial scale	Data were collected over a period of three years, from 2018 to 2020, each year beginning in late January and ending in late June. This is the period during which individuals show territorial behaviour in the study species, which is relevant to record territorial, courtship or self-advertising song as well as breeding data.
Data exclusions	From the observational dataset, no data were excluded from the analysis. From the experimental data set, two trials were removed. One because we noticed disrupted behaviour from the territorial male and another because the test subject did not produce any vocalization during the entire trial.
Reproducibility	Experimental design was simplified and is reported with detail to be potentially replicated. Previous pilot trials were conducted to achieve a reproducible design. During the actual experiment, only two of our trials were unsuccessfull from 15 attempts
Randomization	In our experiment, all individuals received both treatments.
Blinding	During the recording and analysis of birdsong, clutch size was unknown to the observer preventing bias in collecting, measuring or analysing song data. During the analysis of female response in the playback experiment, the observer was blind to experimental treatment. As mentioned before, all subjects received both treatments. To assess female preference we presented songs that varied in consistency, and the observer that measured female vocal response within the audio recordings ignored which of the presented song types had higher or lower vocal consistency, as this was measured only after.

Did the study involve field work? ☒ Yes ☐ No

Field work, collection and transport

Field conditions	Fieldwork was conducted in most weather conditions, except heavy rain and wind, as sound recording devices cannot be used in these conditions.
Location	Lancaster University Campus (54.01′ N, 2.78′ W), 40 masl
Access & import/export	n/a
Disturbance	The observational data involves minimal disturbance to the animals. To assess the impact of our experimental set up on blue tit females, we compared breeding behaviour between the subset of females included in the experiment and the rest of the population. No impact of experimental effect on breeding was detected.

Reporting for specific materials, systems and methods

We require information from authors about some types of materials, experimental systems and methods used in many studies. Here, indicate whether each material, system or method listed is relevant to your study. If you are not sure if a list item applies to your research, read the appropriate section before selecting a response.

Materials & experimental systems

n/a	Involved in the study	
☒	☐	Antibodies
☒	☐	Eukaryotic cell lines
☒	☐	Palaeontology and archaeology
☐	☒	Animals and other organisms
☒	☐	Clinical data
☒	☐	Dual use research of concern
☒	☐	Plants

Methods

n/a	Involved in the study	
☒	☐	ChIP-seq
☒	☐	Flow cytometry
☒	☐	MRI-based neuroimaging

Animals and other research organisms

Policy information about studies involving animals; ARRIVE guidelines recommended for reporting animal research, and Sex and Gender in Research

Laboratory animals	n/a
Wild animals	We studied wild individual blue tits (Cyanistes caeruleus) ranging from 1 year to 8 years of age. These were eventually trapped for marking using mist nets or traps in the nest box. Birds were marked with a combination of leg rings, sexed and aged and released.
Reporting on sex	n/a
Field-collected samples	No field collected samples were used in this study
Ethics oversight	All fieldwork involving blue tits was approved by the Lancaster University animal welfare and ethical review board and licenced, where appropriate, by Natural England, and the British Trust for Ornithology.

Note that full information on the approval of the study protocol must also be provided in the manuscript.
